# Nomogram Predicting Survival to Assist Decision-Making of Metastasectomy in Patients With Metastatic Renal Cell Carcinoma

**DOI:** 10.3389/fonc.2020.592243

**Published:** 2020-12-07

**Authors:** Kan Wu, Zhihong Liu, Yanxiang Shao, Xiang Li

**Affiliations:** Department of Urology, Institute of Urology, West China Hospital, Sichuan University, Sichuan, China

**Keywords:** metastatic renal cell carcinoma, metastasectomy, nomogram, cancer-specific mortality, cytoreductive nephrectomy

## Abstract

The survival benefit of metastasectomy (MSX) in patients with metastatic renal cell carcinoma (mRCC) remains unclear. A reliable model to predict an individuals’ risk of cancer-specific mortality (CSM) and to identify optimal candidates for MSX is needed. We identified 2,911 mRCC patients who underwent cytoreductive nephrectomy from the Surveillance, Epidemiology, and End Results database (2010–2015). Based on the Fine and Gray competing risks analyses, we created a nomogram to predict the survival of mRCC patients. Decision tree analysis was useful for patient stratification. The impact of MSX was assessed among three different subgroups. Overall, 579 (19.9%) cases underwent MSX. In the entire patients, the 1-, 2-, and 3-year cumulative incidence of CSM were 32.8, 47.2, and 57.9%, respectively. MSX was significantly associated with improved survival (hazard ratio [HR] = 0.875, 95% confidence interval [CI] 0.773–0.991; P = 0.015). Based on risk scores, patients were divided into three risk groups using decision tree analysis. In the low-risk group, MSX was significantly associated with a 12.8% risk reduction of 3-year CSM (HR = 0.689, 95% CI 0.507–0.938; P = 0.008), while MSX was not associated with survival in intermediate- and high-risk groups. We proposed a novel nomogram and patient stratification approach to identify suitable patients for MSX. The newly identified patient subgroup with a low-risk of CSM might benefit more from aggressive surgery. These results should be further validated and improved by the prospective trials.

## Introduction

Kidney cancer comprises approximately 2% of all malignant tumors and ranks 14th among the most common cancers in the world (1, 2). More notably, about 20–30% of patients present with synchronous metastatic disease at the time of diagnosis, with a poor prognosis (3). In the past decade, the use of targeted therapies against the VEGF and mTOR pathways has significantly improved the survival of patients with metastatic renal cell carcinoma (mRCC) (4). With the torrential increase in novel immunotherapies largely making these regimens obsolete, as the first-line treatment for mRCC (5).

In addition to modern systemic therapy, cytoreductive nephrectomy also has important value in treating mRCC, allowing a longer survival for well-selected patients with distant disease (6). Of note, metastasectomy (MSX) may also be thought as a potential treatment option to eliminate a large percentage of the tumor burden and sometimes achieve complete remission of the disease (7, 8). Several published studies have shown that surgical excision of the metastases could prolong survival and could be safely done in mRCC patients, even in the presence of multiple metastases (9, 10). Recently, a large hospital-based study based on the National Cancer Database analysis, also suggested that MSX-treated patients had a prolonged survival time when compared to non-MSX treated patients (11).

Obviously, some patients with distant disease may benefit from MSX, but now it is still unclear which subgroups may benefit from aggressive surgery. Proper patient selection strategy has been poorly explored. Therefore, developing a reliable model to identify ideal candidates for MSX is an important consideration. The nomogram is a convenient tool that can quantify survival prediction and has value in patient risk stratification. It has been proved that the prognostic model could provide precise risk prediction in several cancer entities (12, 13). Although several nomograms have been constructed for predicting survival of kidney cancer patients (14–16). The applicable models for guiding mRCC patient to choose MSX are still scant.

Given this background, we used a population-based database to build a prognostic model as a risk stratification strategy, and further determine a patient subgroup who may be more likely to benefit from MSX among mRCC patients.

## Materials and Methods

### Study Population

Data was derived from the National Cancer Institute’s Surveillance, Epidemiology, and End Results (SEER) database, which collects and publishes cancer-speciﬁc outcomes for approximately 28% of the American population. Using the SEER database, we identified patients diagnosed with metastatic RCC between 2010 and 2015.

Inclusion criteria included: (1) patients with renal cancer labeled as the ICD-O-3 site codes (C649, 8000-8980); (2) patients who underwent cytoreductive nephrectomy; (3) patients with pathological confirmation; (4) patients aged 18–90 years at initial diagnosis. Patients with missing data on race, distant sites (lung, liver, bone, or brain), T stage, N stage, tumor grade, or survival status were excluded from the cohort. Likewise, cases with bilateral tumors, and those with unknown surgical information were also excluded.

Excision of metastatic lesions included surgical resection to distant lymph nodes, regional and distant sites. Patients were grouped based on treatment type: MSX group *versus* non-MSX group.

### Demographic and Clinical Variables

Study variables included age, sex, race, tumor stage, tumor location, distant site, tumor grade, treatment, survival months, vital status, and cause of death (based on the SEER cause-of-death classification).

### Statistical Analysis

Differences in demographic and clinical characteristics were examined using the Chi-square test for categorical variables. A univariate and multivariate Fine and Gray’s competing risk analysis was performed to identify risk factors associated with cancer-specific mortality (CSM) and expressed as a hazard ratio (HR) with 95% confidence interval (CI). Non-kidney cancer-related death was considered as competing risk event for cancer-specific death.

The nomogram for predicting CSM was built in patients who did not receive MSX treatment. The factors with a p value < 0.05 in univariate analysis were further incorporated into multivariate analysis. Independent factors of survival were finally identified using the multivariable competing risks proportional hazards model. A nomogram to predict survival was established based on the factors that included in the final model. Internal validation of the nomogram was performed using the concordance index (C index) value, which was used to estimate the discriminative performance of the model. A calibration curve (1,000 bootstrap resamples) was graphically generated to assess the calibration of the nomogram.

According to the nomogram scores, decision tree analysis (Chi-Square automatic interaction detector) was utilized to identify the cutoff points for building risk classification system. The patients were divided into three prognostic groups: the low-, intermediate-, and high-risk groups.

All statistical analyses were performed with SPSS software version 23.0 (IBM, Armonk, NY, USA) and R software (version 3.5.1; R Foundation). A P value <0.05 in a 2-tailed test was determined as statistical signiﬁcance.

### Ethics Statement

We signed the SEER Research Data Agreement (No. 12587-Nov2019) and further searched the data according to the approved guidelines. The SEER data is available to the public, and patients’ records are anonymous. Therefore, this study was deemed exempt from review by our institutional review board.

## Results

Overall, 2,911 eligible patients with mRCC were enrolled in this study. Among them, 579 (19.9%) cases underwent MSX, while 2,332 (80.1%) patients received non-MSX treatment. The median age of the entire patients at first diagnosis was 61 years (interquartile range [IQR] 54–69 years). The baseline characteristics of all patients are summarized in **Table 1**. Patients with lower T stage and lymph node stage, or those with bone metastases and brain metastases were more likely to receive MSX. However, lung metastases were relatively less in the MSX (44.2%) patients and more in non-MSX group (60.6%).

**Table 1:** Baseline demographic and clinical characteristics of the patients.

Characteristic	No MSX	MSX	*P*
	(n = 2,332)	(n = 579)	value
Age, n (%)			
Median (IQR)	62 (54–69)	61 (53–68)	
≤75 y	2,092 (89.7)	533 (92.1)	0.089
>75 y	240 (10.3)	46 (7.9)	
Sex, n (%)			
Male	1,630 (69.9)	413 (71.3)	0.500
Female	702 (30.1)	166 (28.7)	
Race, n (%)			
White	1,951 (83.7)	496 (85.7)	0.239
Other	381 (16.3)	83 (14.3)	
Laterality, n (%)			
Left	1,256 (53.9)	306 (52.8)	0.663
Right	1,076 (46.1)	273 (47.2)	
T stage, n (%)			
T1	288 (12.3)	80 (13.8)	<0.001
T2	285 (12.2)	94 (16.2)	
T3	1,496 (64.2)	312 (53.9)	
T4	263 (11.3)	93 (16.1)	
N stage, n (%)			
N0	1,634 (70.1)	455 (78.6)	<0.001
N1	698 (29.9)	124 (21.4)	
Bone metastases, n (%)			
No	1,655 (71.0)	384 (66.3)	0.029
Yes	677 (29.0)	195 (33.7)	
Brain metastases, n (%)			
No	2,196 (94.2)	507 (87.6)	<0.001
Yes	136 (5.8)	72 (12.4)	
Liver metastases, n (%)			
No	2,057 (88.2)	507 (87.6)	0.669
Yes	275 (11.8)	72 (12.4)	
Lung metastases, n (%)			
No	918 (39.4)	323 (55.8)	<0.001
Yes	1,414 (60.6)	256 (44.2)	
Grade, n (%)			
Grade 1–2	455 (19.5)	98 (16.9)	0.174
Grade 3	949 (40.7)	258 (44.6)	
Grade 4	928 (39.8)	223 (38.5)	

MSX, metastasectomy; IQR, interquartile range.

**Figure 1:**
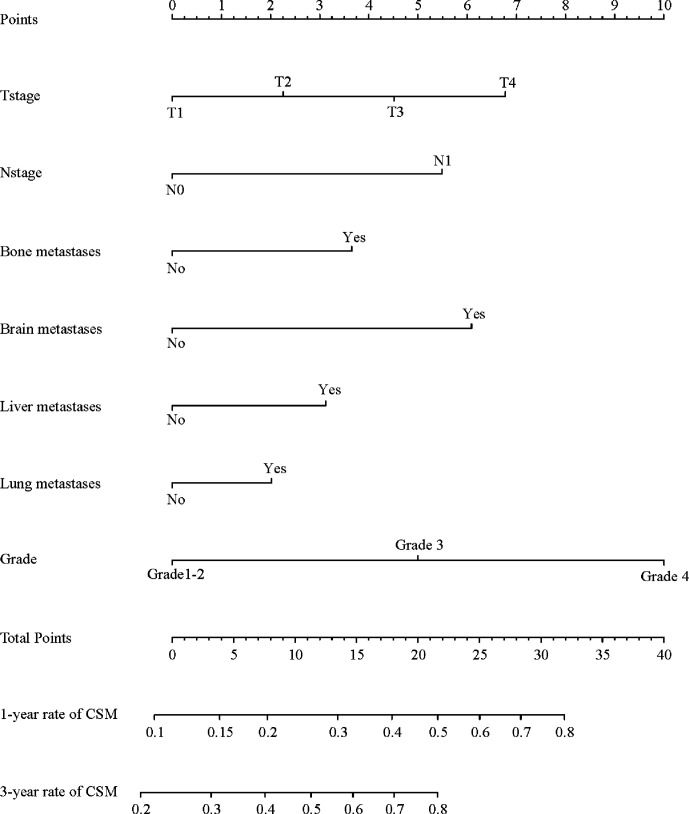
Nomogram predicting CSM for non-MSX treated patients with metastatic kidney cancer. CSM, cancer-specific mortality; MSX, metastasectomy.

Among 2,911 eligible patients, 1,692 (58.1%) patients died of kidney cancer during follow-up period (median time: 17 [IQR 7–32] months). The 1-, 2-, and 3-year cumulative incidence of CSM for all patients were 32.8, 47.2, and 57.9%, respectively. In the non-MSX treated patients, the multivariate competing risks analysis identified T stage, N stage, tumor grade, and distant organs (bone, brain, liver, or lung) as independent factors for CSM (**Table 2**). All confirmed predictors were integrated to develop a prognostic model for survival (**Figure 1**). The C index of the novel nomogram was 0.710, reflecting the good discriminative ability of the predictive model. The calibration plots also showed favorable agreement in the 3-year cumulative incidence of CSM between the nomogram-predicted probabilities and the actual observations (**Supplementary Figure 1**). Each factor in the predictive model was assigned a risk score based on its contribution as presented in the nomogram (**Figure 1**). According to the sum of points, we can easily estimate the predicted probability of CSM for a patient at each time point.

**Table 2:** Univariate and multivariate analysis for cancer-specific mortality in non-MSX treated patients.

Variable	Univariate		Multivariate	
	HR (95% CI)	*P* value	HR (95% CI)	*P* value
Age, y				
≤75	Ref			
>75	1.02 (0.859–1.220)	0.80		
Sex				
Male	Ref		Ref	
Female	1.14 (1.02–1.28)	0.022	1.12 (0.996–1.260)	0.058‬
Race				
White	Ref			
Other	1.07 (0.928–1.220)	0.37		
Laterality				
Left	Ref			
Right	0.92 (0.829–1.020)	0.120		
T stage				
T1	Ref		Ref	
T2	1.50 (1.19–1.90)	<0.001	1.36 (1.07–1.72)	0.011‬
T3	1.91 (1.57–2.32)	<0.001	1.42 (1.16–1.74)	<0.001
T4	2.97 (2.33–3.80)	<0.001	1.90 (1.47–2.46)	<0.001
N stage				
N0	Ref		Ref	
N1	1.84 (1.65–2.05)	<0.001	1.55 (1.38–1.74)	<0.001
Bone metastases				
No	Ref		Ref	
Yes	1.17 (1.04–1.30)	0.007	1.35 (1.20–1.53)	<0.001
Brain metastases				
No	Ref		Ref	
Yes	1.56 (1.25–1.95)	<0.001	1.61 (1.28–2.02)	<0.001
Liver metastases				
No	Ref		Ref	
Yes	1.43 (1.21–1.68)	<0.001	1.25 (1.05–1.49)	0.012
Lung metastases				
No	Ref		Ref	
Yes	1.22 (1.09–1.36)	<0.001	1.19 (1.06–1.34)	0.004
Grade				
Grade1–2	Ref		Ref	
Grade3	1.63 (1.39–1.91)	<0.001	1.42 (1.21–1.68)	<0.001
Grade4	2.71 (2.32–3.18)	<0.001	2.20 (1.86–2.60)	<0.001

MSX, metastasectomy; HR, hazard ratio; CI, confidence interval.

**Figure 2:**
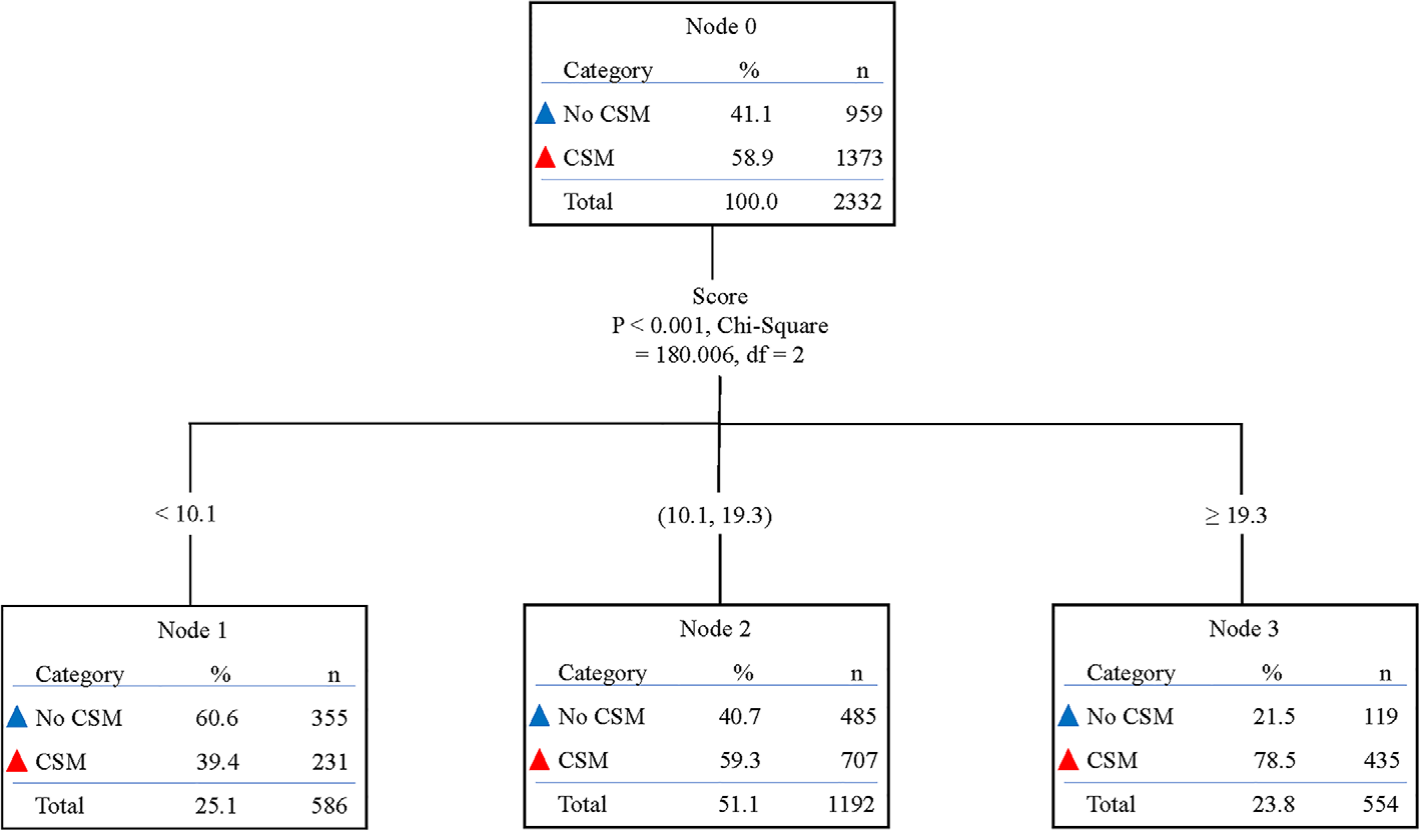
Decision tree analysis according to the effect of nomogram score on cancer-specific mortality in non-MSX treated patients. CSM, cancer-specific mortality; MSX, metastasectomy.

Besides, based on the total risk scores of each patient in the non-MSX group produced by the novel nomogram, we determined two cut-off values (scores 10.1 and 19.3) by using decision tree analysis (**Figure 2**), to build a proper risk classification system. Then, all patients were classified into three prognostic groups: patients with a total score <10.1 were sorted into the low-risk group (n = 744), cases with a total score ≥19.3 were classified into the high-risk group (n = 691), and the remaining patients were assigned to the intermediate-risk group (n = 1,476). In the total cohort, these three risk subgroups had a distinct difference in the 3-year cumulative incidence of CSM: 35.6% in the low-risk group, 59.0% in the intermediate-risk group, and 80.4% in the high-risk group, respectively (P < 0.001; **Supplementary Figure 2**).

**Figure 3:**
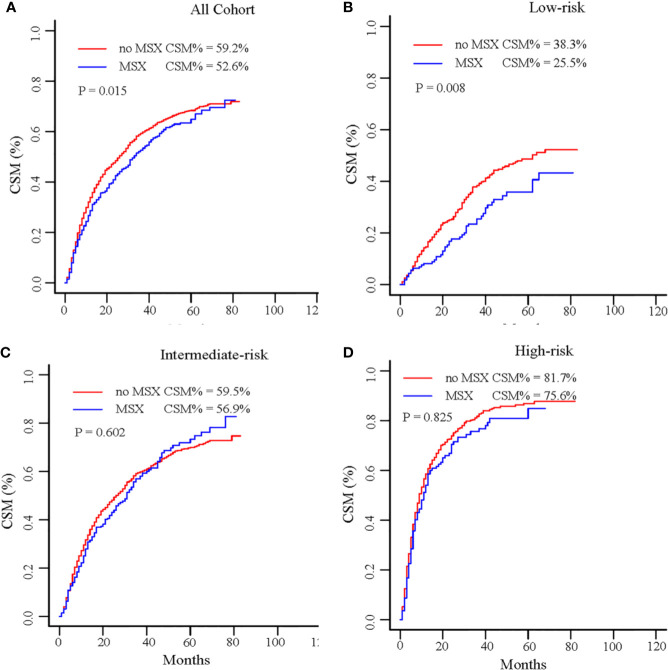
Cumulative incidence of CSM by MSX in the entire cohort **(A)**, low-risk group **(B)**, intermediate-risk group **(C)**, and high-risk group **(D)**. CSM, cancer-specific mortality; MSX, metastasectomy.

For the entire patients, MSX was significantly associated with decreased CSM compared with non-MSX treatment (3-year cumulative incidence 52.6 *vs* 59.2%; HR = 0.875, 95% CI 0.773–0.991; P = 0.015; **Table 3**; **Figure 3**). In the subgroup analysis (**Table 3**; **Figure 3**), MSX was also correlated with a decrease in CSM in the low-risk group (3-year cumulative incidence 25.5 *vs* 38.3%; HR = 0.689, 95% CI 0.507–0.938; P = 0.008). However, MSX was not correlated with reduced CSM in the intermediate-risk group (3-year cumulative incidence 56.9 *vs* 59.5%; HR = 0.972, 95% CI 0.823–1.149; P = 0.602), and high-risk group (3-year cumulative incidence 75.6 *vs* 81.7%; HR = 0.825, 95% CI 0.661–1.030; P = 0.825).

**Table 3:** Effect of MSX on cancer-specific mortality according to multivariate analysis in different groups.

	Treatment	3-year cumulative incidence of CSM, %	*P* value	HR (95% CI)
All patients (n = 2,911)	MSX	52.6	0.015	0.875 (0.773–0.991)
	no MSX	59.2		
Low-risk (n = 744)	MSX	25.5	0.008	0.689 (0.507–0.938)
	no MSX	38.3		
Intermediate risk (n = 1,476)	MSX	56.9	0.602	0.972 (0.823–1.149)
	no MSX	59.5		
High risk (n = 691)	MSX	75.6	0.185	0.825 (0.661–1.030)
	no MSX	81.7		

MSX, metastasectomy; CSM, cancer-specific mortality; HR, hazard ratio; CI, confidence interval.

HR in multivariate analysis were adjusted for age, sex, race, tumor stage, tumor location, metastatic sites, tumor grade.

## Discussion

Advances in contemporary systemic therapy, specifically since the introduction of targeted therapy, have shown a remarkable improvement in survival for patients with mRCC. However, it’s relatively rare to obtain complete remission with targeted agents alone (17), and many patients may suffer from side effects associated with pharmacotherapy. Moreover, the economic burden for mRCC patients is relatively heavy (18). Conversely, the survival benefit from MSX has shown encouraging results, providing a chance to achieve complete remission of the disease and improved survival (19). According to the study by Brecheteau et al., all 22 mRCC patients who experienced a complete remission were treated with cytoreductive nephrectomy. Among them, 21 patients underwent surgery for distant metastases (7).

However, the role of MSX in patients with mRCC remains unclear and has not been well-studied in the era of targeted therapy. Several previous studies on MSX in the targeted therapy era have shown the safety and feasibility of MSX in mRCC patients, and provided a long-term complete remission and favorable prognosis (20–22). However, these studies were considerably constrained by a small number of cases and only a specific group, which resulted in inhibiting their generalization. Indeed, given the absence of prospective clinical trials evaluating the role of MSX, large-scale cohort studies could be used to examine the value of MSX in patients with metastatic disease. A SEER-based analysis by Palumbo (23) showed that metastasectomy was associated with lower overall mortality risk (median survival 11 *vs.* 9 months, P = 0.002). Recently, a large hospital-based national study found that the significant increases in MSX utilization for metastatic kidney cancer, and patients undergoing MSX did benefit from a prolonging survival, with a 17% risk reduction of death, highlighting the potential value of MSX in the treatment management of mRCC (11). Similarly, in this population-based investigation, we found that MSX was significantly associated with a 6.6% risk reduction in the 3-year cumulative incidence of CSM for patients in the whole mRCC cohort, which further indicated that MSX is of great importance in improving the prognosis of patients with distant disease.

The key issue to consider next is to better understand how clinicians make a decision rationale in selecting patients to undergo MSX or not. In the present study, based on patients’ clinicopathological characteristics, we established a novel nomogram and risk classification system for predicting individuals’ risk of CSM, which can be useful in aiding decision-making of MSX in patients with mRCC. Our finding revealed that patients with favorable biological profile at a lower risk of CSM were more likely to benefit from MSX, with a 12.8% risk reduction of kidney cancer-specific death. However, MSX did not confer any survival advantage in the intermediate-risk, and high-risk groups compared with non-MSX treated patients. Likewise, data from a previous study suggested that the selected patients with favorable risk might benefit from MSX and achieve better survival (24). Notably, the first prospective phase III trial (CARMENA) in the targeted therapy era also showed that mRCC patients with intermediate and poor-risk could not benefit from cytoreductive nephrectomy (25). Therefore, the present predictive model can be used to improve the understanding of the potential role of MSX in the metastatic scenario, and has value in clinical decision-making, patient counseling, and clinical trial design.

From a clinical standpoint, our study showed that MSX could confer a survival benefit for the low-risk patients, rather than patients with an unfavorable oncological profile at higher risk of CSM, thereby avoiding over- or undertreatment for patients. In addition, all predictive factors included in the prognostic model can be easily extracted from clinical practices. Meanwhile, the C index of the present nomogram for predicting survival was 0.710, which is similar to the International Metastatic Renal Cell Carcinoma Database Consortium (IMDC) model (C index = 0.70) (16), reflecting its great performance and favorable clinical effect. However, there are many elusive issues on this topic. For one, how to develop a multimodal strategy for mRCC patients by combining MSX with drug therapy. Given the disparate patterns of treatment modalities (26), and the lack of high-level evidence, current guidelines do not include specific recommendations on patient selection in the setting of mRCC (27). Notably, the important upside of our finding is that the potential benefits of MSX depend on patient’s health conditions and tumor characteristics.

In addition to the prediction model for survival, in the present study, risk classification is another pivotal step to identify suitable subgroups for MSX in patients with mRCC. Using decision tree analysis, we developed a proper risk classification strategy, which could satisfactorily separate all patients into three different risk groups with distinct CSM rates. Therefore, our approach included predictive model and decision tree analysis, and demonstrated its great performance in patient stratification, enabling clinicians to conveniently estimate individuals’ risk of CSM and select suitable patients who are more likely to benefit from MSX. This approach may be a meaningful and practical tool for helping tailor individualized treatment.

There are certain limitations in our study. First, although this study based on SEER database covers approximately 28% of the American population, the sample size of MSX group is relatively small, and is limited by the inherent bias of retrospective analysis. Second, there is a lack of information on patient performance status, comorbidities, complications of MSX, which may be used in treatment decision-making. Third, detailed information about targeted therapy and other distant sites are unavailable in the SEER database, which undoubtedly affect the survival of mRCC patients. Fourth, although the information of the metastatic organs was recorded, it was not clear that surgery was performed at that site, and we could not determine whether the patients underwent complete resection. Finally, in this cohort, we could not assess whether patients experienced recurrences or metachronous metastases after MSX. Despite these limitations, we first developed a prognostic nomogram for mRCC to identify suitable patients who could benefit from MSX, which can be conveniently perform in clinical practice.

## Conclusion

Using the prognostic nomogram and decision tree analysis, we proposed a new approach for individuals’ risk prediction and corresponding risk classification system to identify optimal candidates for MSX among patients with mRCC. We found that patients at low risk of CSM might benefit more from MSX. Conversely, MSX did not confer any survival advantage in intermediate-, or high-risk patients. As a novel model, there is a definite need for a prospective trial to validate and improve the merits of our model.

## Data Availability Statement

The original contributions presented in the study are included in the article/**Supplementary Material**. Further inquiries can be directed to the corresponding author.

## Ethics Statement

Ethical review and approval was not required for the study on human participants in accordance with the local legislation and institutional requirements. Written informed consent for participation was not required for this study in accordance with the national legislation and the institutional requirements.

## Author Contributions

KW wrote the draft of the article, did the literature search, and contributed to data collection and analysis. ZL wrote the draft of the article and did the figure editing. YS contributed to data collection and analysis. XL contributed to data interpretation and corrections to the article. All authors contributed to the article and approved the submitted version.

## Funding

This work was supported by the 1.3.5 project for disciplines of excellence, West China Hospital, Sichuan University.

## Conflict of Interest

The authors declare that the research was conducted in the absence of any commercial or financial relationships that could be construed as a potential conflict of interest.

## Supplementary Material

Supplementary Figure 1 Calibration curves showing the probability of CSM between the nomogram prediction and the actual observation. A plot along the 45-degree line would indicate a perfect calibration model in which the predicted probabilities are identical to the actual outcomes. CSM, cancer-specific mortality.

Supplementary Figure 2 Cumulative incidence of CSM for the entire patients in the low-, intermediate-, and high-risk groups. CSM, cancer-specific mortality.
