# Supplementary figures and images for: Nomogram Predicting Survival to Assist Decision-Making of Metastasectomy in Patients With Metastatic Renal Cell Carcinoma
Source: Front Oncol. 2020 Dec 7;10:592243. doi: 10.3389/fonc.2020.592243 (PMC7793951; doi:10.3389/fonc.2020.592243)

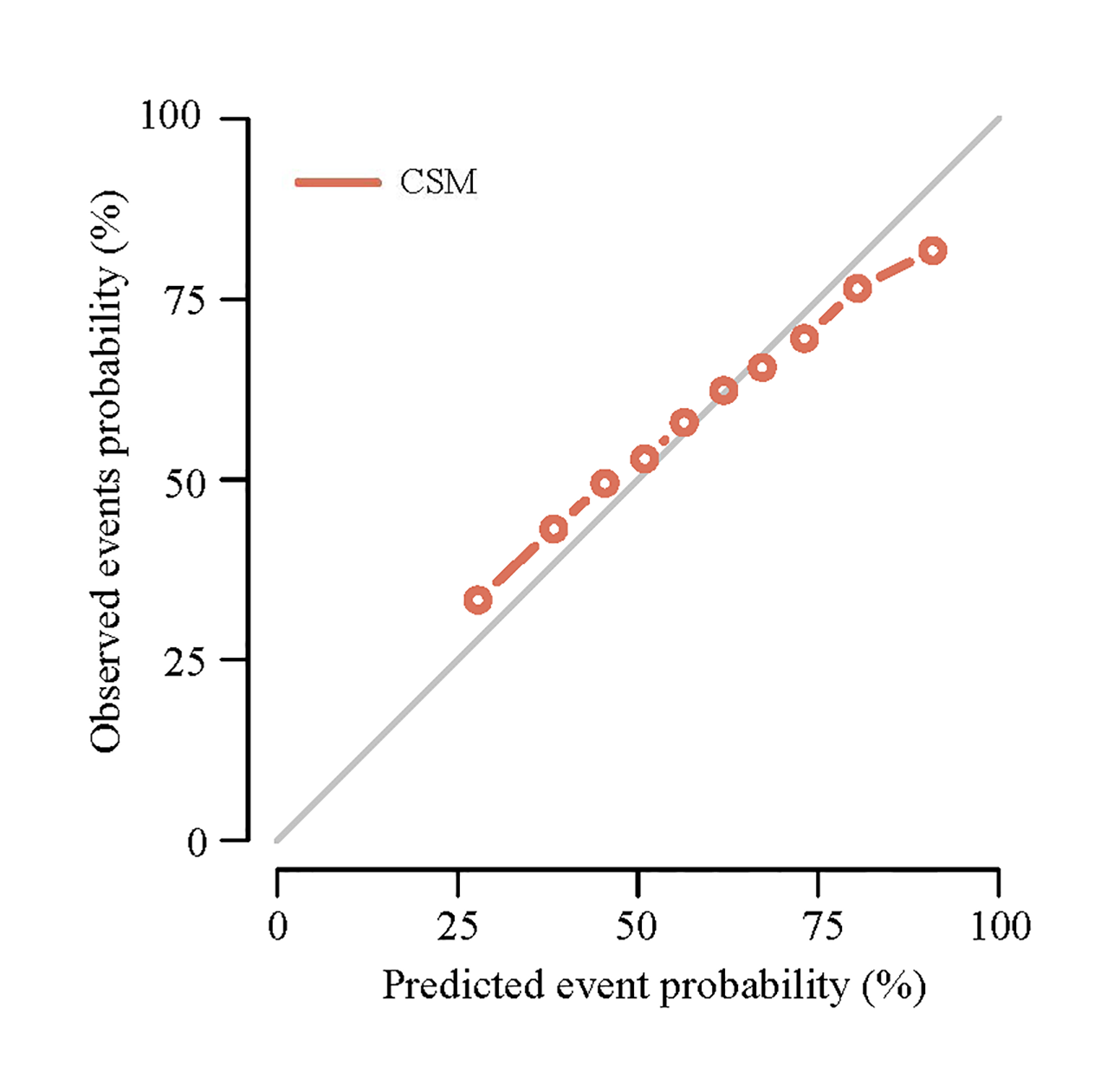

Supplement: Supplementary Figure 1 — Calibration curves showing the probability of CSM between the nomogram prediction and the actual observation. A plot along the 45-degree line would indicate a perfect calibration model in which the predicted probabilities are identical to the actual outcomes. CSM, cancer-specific mortality. [file Image_1.tif]

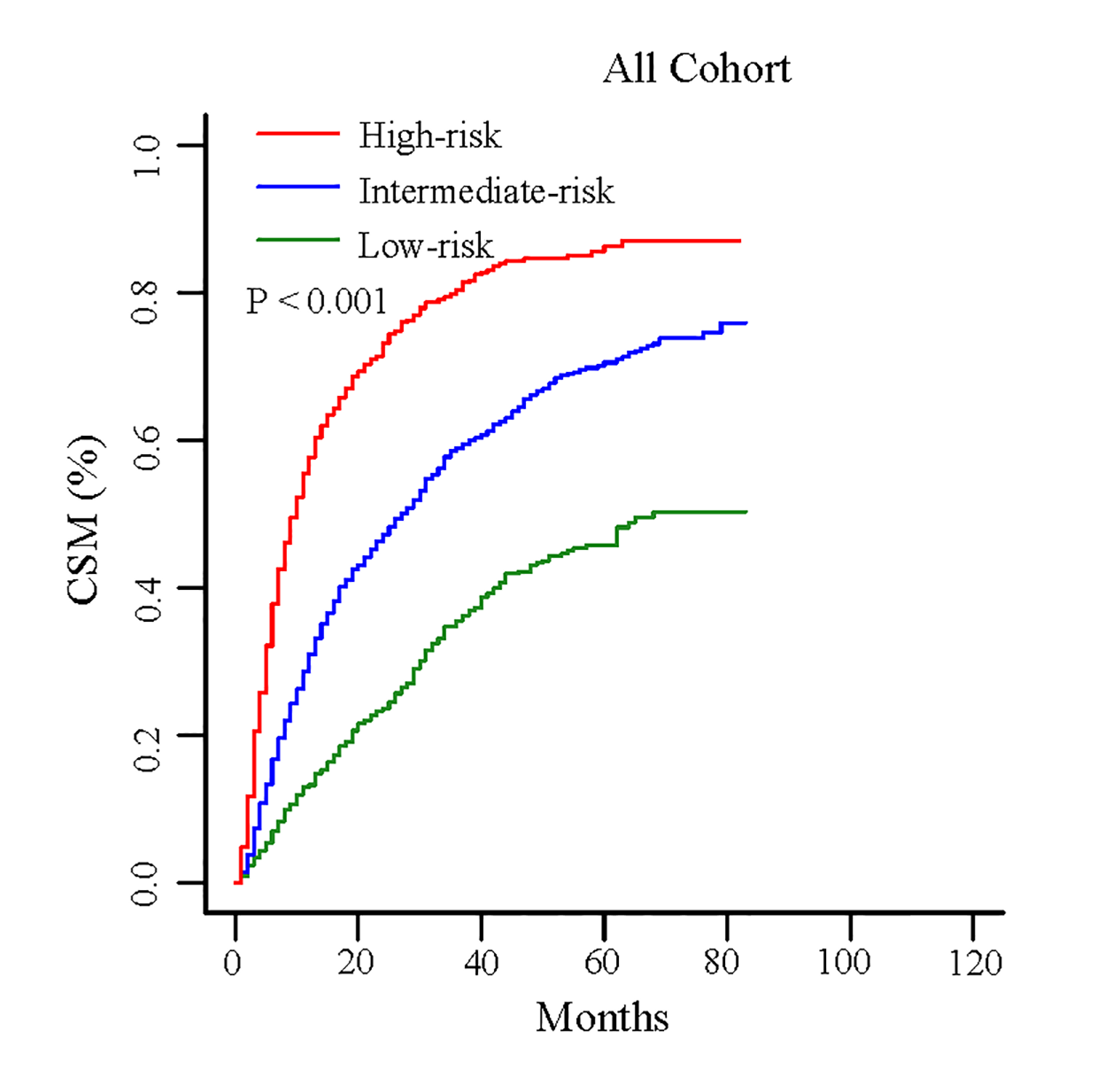

Supplement: Supplementary Figure 2 — Cumulative incidence of CSM for the entire patients in the low-, intermediate-, and high-risk groups. CSM, cancer-specific mortality. [file Image_2.tif]
